# Do All Integrase Strand Transfer Inhibitors Have the Same Lipid Profile? Review of Randomised Controlled Trials in Naïve and Switch Scenarios in HIV-Infected Patients

**DOI:** 10.3390/jcm10163456

**Published:** 2021-08-04

**Authors:** Maria Saumoy, Jose Luís Sanchez-Quesada, Jordi Ordoñez-Llanos, Daniel Podzamczer

**Affiliations:** 1 HIV and STD Unit, Infectious Disease Department, Bellvitge University Hospital, C/Feixa Llarga s/n, L’Hospitalet de Llobregat, 08907 Barcelona, Spain; dpodzamczer@bellvitgehospital.cat; 2 Bellvitge Institute for Biomedical Research, L’Hospitalet de Llobregat, 08907 Barcelona, Spain; 3 Cardiovascular Biochemistry Group, Biomedical Research Institute IIB Sant Pau, 08041 Barcelona, Spain; jsanchezq@santpau.cat (J.L.S.-Q.); JOrdonez@santpau.cat (J.O.-L.); 4 Foundation for the Biochemistry and Molecular Pathology, 08041 Barcelona, Spain

**Keywords:** integrase strand transfer inhibitors, antiretroviral therapies, HIV, lipid profile, randomised controlled trials

## Abstract

In this study, we aim to explore the effects on lipids of integrase strand transfer inhibitors (INSTIs) in naïve and switch randomised controlled trials, and compare them with protease inhibitors (PIs) and non-nucleoside reverse transcriptase inhibitors (NNRTIs). We reviewed phase 3/4 randomised clinical trials in the Cochrane and PubMed databases that compare an INSTI with a boosted PI, an NNRTI, or another INSTI plus one or two nucleoside/nucleotide reverse transcriptase inhibitors (NtRTIs) in naïve patients and switching strategies in HIV-infected patients. We reported the baseline plasma concentration of total cholesterol (TC), low and high-density lipoprotein cholesterol (LDL-c, HDL-c), triglycerides (TG), and the TC/HDL-c ratio, as well as the change at weeks 48 and 96, when available. In naïve HIV-infected patients, raltegravir (RAL) and dolutegravir (DTG) have a more favourable lipid profile compared with NNRTI and boosted PI. Elvitegravir (EVG/c) has a superior lipid profile compared with efavirenz and is similar to that observed with ritonavir-boosted atazanavir except in TG, which increases less with EVG/c. In naïve patients, RAL, DTG, and bictegravir (BIC) produce a similar, slight increase in lipids. In switching trials, the regimen change based on a boosted PI or efavirenz to RAL, DTG, or BIC is associated with clinically significant decreases in lipids that are minor when the change is executed on EVG/c. No changes were observed in lipids by switching trials between INSTIs. In summary, RAL, DTG, and BIC have superior lipid profiles compared with boosted-PI, efavirenz, and EVG/c, in studies conducted in naïve participants, and they are associated with a clinically significant decrease in lipoproteins by switching studies.

## 1. Introduction

Since the introduction of combined antiretroviral therapy (ART) in 1996, there has been a decrease in the global mortality of people living with human immunodeficiency virus type 1 (HIV-1; PLWH), mainly concerning deaths related to acquired immunodeficiency syndrome (AIDS). However, the proportion of deaths from non-AIDS-related disorders, such as cardiovascular (CV), non-AIDS cancer, and liver diseases, has increased [1]. The risk of myocardial infarction is one and a half- to two-fold greater in PLWH compared with the general population, and is similar to diabetes mellitus, hypertension, or smoking [2]. The increased CV risk in PLWH is due to the addition of several factors, such as the effects of HIV through inflammation and immune activation that are not completely reverted by combined ART, the higher frequency of traditional CV risk factors in PLWH, and the deleterious effects of some antiretroviral drugs on lipid and glucose metabolism [3]. Several factors may interact, favouring the presence of lipid disorders in PLWH, among them, HIV, lifestyle factors (including diet and exercise), genetic factors, and ART [4]. 

Non-treated chronic HIV infection, as well as other chronic infections and inflammation conditions, is associated with an atherogenic lipid phenotype. This phenotype is characterized by changes in lipid concentrations, including decreased levels of high-density lipoprotein cholesterol (HDL-c), total cholesterol (TC), and increased levels of triglycerides (TG), as well as modifications in lipid composition and function [5,6,7,8]. The initiation of combined ART has been associated with lipid changes, which can be considered a return to a “healthy” state [5,8]. 

ART has evolved rapidly since 1986, when the first active drug against HIV, zidovudine, a nucleoside reverse transcriptase inhibitor (NRTI), became available. Since then, new drugs from the same family and new families of drugs have been incorporated into the HIV therapeutic arsenal (Table 1). In 2007, the integrase strand transfer inhibitor (INSTI) family joined the arsenal of HIV treatment. Raltegravir (RAL) was the first to be approved, followed by cobicistat-boosted elvitegravir (EVG/c), dolutegravir (DTG), bictegravir (BIC), and recently, cabotegravir. INSTIs have proven effective and safe, with a good lipid profile, for treatment-naïve patients and switching strategies in HIV-1-infected patients, which is why DTG and BIC are guideline-preferred regimens for the initial treatment of PLWH and are largely used in switching strategies to improve toxicities [9,10]. 

Combined ART consists of a backbone of two nucleoside/nucleotide reverse transcriptase inhibitors (NtRTIs) and a third drug that may be an INSTI, a boosted protease inhibitor (PI), or a non-nucleoside reverse transcriptase inhibitor (NNRTI) [9,10]. Lipid disorders associated with ART differ significantly depending on the family and within the same family. Old PIs, NRTIs, and some NNRTIs are the agents most frequently related to increases in TG, TC, and low-density lipoprotein cholesterol (LDL-c). The management of lipid disorders has been an important issue in the treatment of PLWH, and is based on guidelines used in the general population, as well as the possibility of switching any component of the ART combination to another with a superior lipid profile [11]. Current guidelines consider switching a boosted PI or efavirenz (EFV)-based regimen to INSTI in patients with dyslipidemia or CV events [9,10]. The objective of this review is to explore the effects on lipids of INSTI compared with PIs and NNRTIs and examine whether the lipid profile differs among INSTIs in naïve and switch randomised controlled trials (RCTs). 

## 2. Materials and Methods

In the Cochrane and PubMed databases, we reviewed RCTs that compared a regimen including an INSTI with a regimen including another INSTI, a boosted PI, or an NNRTI, plus one or two NtRTIs, recommended in past and current guidelines [9,10]. From the selected RCTs, we included phase 3/4 RCTs conducted in naïve HIV-infected patients and switch trials where the PI, NNRTI, or INSTI of a combined ART regimen was changed to an INSTI in virologically suppressed HIV-infected patients. We excluded RCTs conducted in special populations, such as pregnant women, children, or those using drug doses other than those approved [9,10]. We also excluded trials without data on plasma lipids.

We reported the baseline TC, LDL-c, HDL-c, TG, and the TC/HDL-c or HDL-c/TC ratio in each arm, when available. We also reported the mean (standard deviation [SD]) or median (interquartile range [IQR]) change from baseline to week 48 for each variable. When available, we described the changes at week 96 to assess the long-term effects of INSTIs. Data are presented in mg/dL. Since the study design, participants, interventions, and reported outcome measures vary markedly, we focused on describing the studies and their results rather than meta-analysing them.

## 3. Results and Discussion

### 3.1. Comparison of INSTIs with Other ART Families in Treatment-Naïve Patients

#### 3.1.1. Raltegravir

In the STARTMRK trial, RAL was compared with EFV in combination with tenofovir disoproxil fumarate (TDF) and emtricitabine (FTC). At week 48, there was a greater increase in the EFV arm than in the RAL arm in terms of TC, LDL-c, HDL-c, and TG (*p* ≤ 0.001 for all comparisons except for LDL-c, where *p* = 0.002), whereas the HDL-c/TC ratio did not change in any arm [12]. At week 96, the mean change from baseline decreased in both arms compared with week 48, but persisted at a significantly higher level in the EFV arm compared with the RAL arm in terms of TC, LDL-c, HDL-c, and TG (*p* < 0.001 for each comparison). The HDL-c/TC ratio remained unchanged in both arms. Lipid-lowering therapy was used in 7% of subjects with the RAL arm and 9% of subjects with the EFV arm at some point through week 96 (Table 2) [13].

The AIDS Clinical Trials Group (ACTG) A5257 trial compared RAL, ritonavir-boosted darunavir (DRV/r), and atazanavir (ATZ/r) combined with TDF and FTC. At week 48, there was a greater increase in DRV/r and ATZ/r compared with the RAL arm in TC, LDL-c, and TG (*p* ≤ 0.001 for all comparisons). At week 96, similar mean changes from baseline were found in the three arms in terms of TC, LDL-c, and TG (all *p* ≤ 0.001). HDL-c increased modestly, with no significant differences between the three arms. From baseline to week 96, the percentage of participants who received lipid-lowering therapy increased from 6% to 14% in the DRV/r arm, 5% to 11% in the ATZ/r arm, and 6% to 9% in the RAL arm [14], respectively (Table 2).

#### 3.1.2. Elvitegravir

The co-formulated EVG/c, TDF, and FTC was compared with the co-formulated EFV, TDF, and FTC. At week 48, median concentrations of TC, LDL-c, and HDL-c increased less in the EVG/c arm (9.6, 10, and 5, respectively) than in the EFV arm (17.4, 16.8, and 7.7, respectively; *p* ≤ 0.001). Changes in the TC/HDL-c ratio and TG were similar in both groups [15].

The co-formulated EVG/c, TDF, and FTC were also compared with ATZ/r plus TDF and FTC. At week 48, the median (IQR) increases in TC, LDL-c, and HDL-c did not differ between arms; these increases were 10 (−6.29), 10.8 (−4.25), and 5.8 (0.12), respectively, in the EVG/c arm and 8 (−12.30), 10.4 (−8.27), and 5 (2.11) for the same variables in the ATZ/r arm. TG increased more in the ATZ/r arm compared with the EVG/c arm (23 (−11.59) vs. 8 (−19.37); *p* = 0.006] [16]. At week 96, there was a significant increase (*p* = 0.046) in median TC in the EVG/c arm (14 (−3.31)) compared with that of the ATZ/r arm (8 (−12.30)). By contrast, TG had a greater increase in the ATZ/r arm compared with the EVG/c arm (16 (−13.52) vs. 5 (−22.37); *p* = 0.012). There were no significant changes from baseline through week 96 for LDL-c, HDL-c, and the TC/HDL-c ratio in either treatment arm [17].

The WAVES trial compared the co-formulated EVG/c, TDF, and FTC with ATZ/r, TDF, and FTC in women. The median (IQR) changes at week 48 in terms of TC, LDL-c, HDL-c, and TG were slight and similar between groups. The levels were 7 (−7.25), 0 (−13.14), 4 (−3.11), and 5 (−16.27) in the EVG/c arm, and 2 (−14.20), −2 (−15.11), 3 (−3.10), and 8 (−18.35) in the ATZ/r arm. The median change in TC/HDL-c ratio was −0.1 in both groups [18].

#### 3.1.3. Dolutegravir

The SINGLE trial compared DTG, abacavir (ABC), and lamivudine (3TC) with the co-formulated EFV, TDF, and FTC. At week 48 in the EFV arm, there was a greater increase in mean TC (*p* = 0.005) and LDL-c (*p* = 0.032); TG and HDL-c showed a similar increase in both arms. The median change in the TC/HDL-c ratio was −0.1 in both arms [19,20]. Small increases in lipid variables were observed in both arms from weeks 48 to 96 (Table 2) [21].

The FLAMINGO trial compared DTG and DRV/r combined with TDF and FTC (67%) or ABC and 3TC (33%). At week 48, the mean increase in TC, LDL-c, and TG was greater in the DRV/r arm compared with the DTG arm. The mean increase in HDL-c was small, slight and comparable between the two arms, whereas the TC/HDL-c ratio increased only in the DRV/r arm (Table 2) [20,22]. 

A fixed-dose combination of DTG, ABC, and 3TC was compared with ATZ/r plus TDF, and FTC in the ARIA trial for women. There were no significant differences between the two treatment arms in the mean change from baseline in TC/HDL-c ratio and TG [23].

The ADVANCE trial compared DTG and FTC plus either tenofovir alafenamide (TAF) or TDF against the co-formulated EFV, TDF, and FTC. The study was conducted in South Africa. At week 48, small changes in both regimens containing DTG were favourable to the TDF-containing regimen (*p* < 0.05 for all variables, except HDL-c at *p* = 0.05). In the EFV arm at week 48, there was a greater increase in TC and HDL-c compared with the DTG-TAF-based regimen (*p* < 0.05) and in all lipid variables compared with the DTG-TDF-based regimen (*p* < 0.001, except TG, at *p* = 0.0058) (Table 2) [24].

**Table 2:** Comparison of INSTIs with other ART families in treatment-naïve patients.

Trial, Year [Ref] Design of the Study	Treatment Arm *n* (Participants); Age; % Men	Study Period	Total Cholesterol	LDL-c	HDL-c	Triglycerides	HDL-c/TC or TC/HDL-c	% on Lipid-Lowering Therapy
**STARTMRK trial** 2009 [12] 2010 [13] Double-blind	**Raltegravir, TDF, FTC**, *n* = 281;37.6; 81%	Baseline ^1^	342 (75.7)	208.1 (67.6)	82.2 (27)	613.3 (361.1)	0.25 (0.08) ^a^	
		Week 48 ^2^	21.2 (62.5)	12.7 (52.9)	8.9 (18.1)	−14.2 (400)	−0.02 (0.06) ^a^	
		Week 96 ^3^	10	7	3	−4		7%
	**Efavirenz, TDF, FTC**, *n* = 282; 36.9; 82%	Baseline ^1^	333.9 (82.2)	198.5 (65.6)	81.1 (23.9)	669.9 (606.2)	0.24 (0.08) ^a^ −0.01 (0.08) ^a^	
		Week 48 ^2^	70.3 (72.2) *	34.4 (62.2) *	21.6 (23.5) *	184.1 (633.6) *		
		Week 96 ^3^	38 *	21 *	10 *	40 *		9%
**ACTG A5257** 2015 [14] Open-label	**Raltegravir, TDF, FTC**, *n* = 600; 37; 76%	Baseline ^4^	158.3 (155,161)	94.9 (92,97)	39.5 (38,41)	123.4 (117,130)		6%
		Week 48 ^5^	159.5 (156,162) [1.2]	92.2(90,95) [−2.9]	44.5 (43,46)	115.3 (109,122) [−7.1]		
		Week 96 ^5^	163.4 (160,166) [5.2]	92 (90,94) [0.1]	45.4 (44,47)	116.3 (110,123) [−7.1]		9%
	**DRV/r, TDF, FTC**, *n* = 595; 37.5; 76%	Baseline ^4^	157 (154,160)	93 (90,95)	40.4 (39,41)	124.3 (117,131)		6%
		Week 48 ^5^	172 (169,176) [15.3] *	99.1 (96,102) [6.1] *	46 (45,47)	137.3 (130,145) [16.8] *		
		Week 96 ^5^	172 (169,176) [15.4] *	99.9 (97,103) [5.1] *	46 (44,47)	141.1 (131,151) [16.8] *		14%
	**ATZ/r, TDF, FTC**, *n* = 602; 37.6; 76%	Baseline ^4^	156.7 (154,159)	93.7 (91, 96)	38.8 (38,40)	123.8 (117,130)		5%
		Week 48 ^5^	169.8 (166,173) [13.1] *	97.4 (94,100) [3.7] *	45.1 (44,46)	139.7 (132,147) [17.1] *		11%
		Week 96 ^5^	172.3 (169,176) [15.3] *	99.4 (96,102) [6.4] *	45.2 (44,46)	140.9 (133,149) [17.1] *		
**SINGLE** 2013 [19] Double-blind	**DTG, ABC, 3TC**; *n* = 414; 35; 84%	Baseline ^1^ Week 48 ^2^	158.9 (34) 17.1 (26)	93.1 (29) 8.5 (21)	43.4 (13) 5.2 (9)	115 (78) 17.7 (94)	3.9 (1) −0.1 (1)	
	**Efavirenz, TDF, FTC** *n* = 414; 35; 84%	Baseline ^1^ Week 48 ^2^	158.2 (37) 24.1 (34) *	92.7 (22) 13.1 (30) *	43.6 (13) 8 (11)	111.2 (67) 18.6 (92)	3.9 (1) −0.1 (1)	
**FLAMINGO** 2014 [22] Open-label	**DTG, TDF, FTC or ABC, 3TC** *n* = 242; 34; 87%	Baseline ^1^ Week 48 ^2^	157.6 (33) 4.3 (24)	91.1 (29) 3.1 (20)	43.9 (13) 2 (9)	114 (66) −5.5 (53)	3.9 (1) 0 (1)	
	**DRV/r, TDF, FTC or ABC, 3TC** *n* = 242; 34; 83%	Baseline ^1^ Week 48 ^2^	162.5 (35) 22.5 (33)	95.5 (29) 14.1 (25)	43.5 (13) 2.2 (10)	117.9 (67) 33.1 (73)	4.1 (2) 0.4 (1)	
**ADVANCE** 2019 [24] Open-label	**DTG, TAF, FTC** *n* = 351; 33; 39%	Baseline ^6^ Week 48 ^7^	146.7 (69,297) 3.9 (−162,212) †	88.8 (15,185) 3.9 (−96,66) †	42.5 (4,100) 3.9 (−50,89)	79.6 (26,433) 0 (−221,956) †		
	**DTG, TDF, FTC**; *n* = 351; 32; 41%	Baseline ^6^ Week 48 ^7^	142.9 (69,251) −3.9 (−127,73)	88.8 (27,224) 0 (−116,69)	42.5 (12,100) 3.9 (−31,58)	70.8 (26,372) −8.8 (−230,487)		
	**Efavirenz, TDF, FTC**; *n* = 351; 32; 43%	Baseline ^6^ Week 48 ^7^	142.9 (54,259) 11.6 (−120,127) ††	99.7(27,235) 3.9 (−131,85) ††	42.5 (8135) 11.3 (−31,85) ††	79.6 (26,451) 0 (−319,354) ††		

Only studies showing baseline data are shown. * *p*-value < 0.05 for comparisons between treatment arms. † *p*-value < 0.05 for the comparison between DTG, TAF, FTC, and DTG, TDF, FTC. †† *p*-value < 0.05 for the comparison between EFV and DTG containing regimens. ^a^ HDL-c/TC ratio. Superindex numbers indicate the statistical parameters to express data: ^1^ Mean (standard deviation); ^2^ Mean change (standard deviation); ^3^ Mean change; ^4^ Mean absolute value (95% confidence interval); ^5^ Mean absolute value (95% confidence interval) [mean change]; ^6^ Median (interquartile range); ^7^ Median change (interquartile range). Abbreviations: ABC, abacavir; ATZ/r, ritonavir-boosted atazanavir; DRV/r, ritonavir-boosted darunavir; DTG, dolutegravir; FTC, emtricitabine; TAF, tenofovir alafenamide; TDF, tenofovir disoproxil fumarate; 3TC lamivudine.

#### 3.1.4. Summary of Studies Comparing INSTI-Based Regimens with Other ART Families in Treatment-Naïve Patients

RAL and DTG were associated with a lower increase in TC, LDL-c, and TG compared with efavirenz and PI/r-based regimens. By contrast, HDL-c increased more in EFV-based regimens. INSTI-based regimens were associated with no increase in the TC/HDL-c ratio. In the STARTMRK trial, the high baseline plasma lipid concentrations were notable, as well as their increase at week 48 in both the RAL and EFV arms. Elvitegravir, the only cobicistat-boosted INSTI, was associated with an intermediate effect on lipid plasma concentrations between the other INSTIs and PI/r or EFV. Cobicistat is a CYP3A4 inhibitor that shows a lower potential for worsening lipid metabolism compared with ritonavir other in vitro studies [25]. However, when combined with EVG, it has a slightly worse metabolic profile compared with non-enhancer INSTIs. 

### 3.2. Comparison of Different INSTIs Strategies in Treatment-Naïve Patients 

#### 3.2.1. Elvitegravir

Elvitegravir/c plus either TAF and FTC or TDF and FTC were compared. At week 48, the median increase in TC, LDL-c, HDL-c, and TG was greater—almost double—in the TAF-containing regimen (*p* < 0.001 for all variables except TG, *p* = 0.027), whereas a slight, similar increase (*p* = 0.84) was observed in both arms in the TC/HDL-c ratio. Thirty-one (3.6%) participants in the TAF-containing arm and twenty-five (2.9%) in the TDF-containing arm started lipid-lowering therapy (*p* = 0.42) (Table 3) [26].

#### 3.2.2. Dolutegravir and Bictegravir

DTG was compared with BIC, both combined with TAF and FTC, in the GS-US-380-1490 study. There were no differences in the median, mostly increasing changes of lipid parameters from baseline at weeks 48 and 96 in both arms. Lipid-lowering therapy was initiated at weeks 48 and 96 in 2% and 3% of cases in the BIC arm, and 2% and 4% in the DTG arm, respectively (Table 3) [27,28].

DTG combined with ABC and 3TC was compared with BIC combined with TAF and FTC in the GS-US-380-1489 trial. There were no differences in the median changes of lipid variables from baseline at week 48, except a small but statistically significant decrease of TC/HDL-c ratio in the DTG arm (*p* = 0.0130). During the study, initiations of lipid-lowering therapy were 2.5% and 2.9% in the BIC and DTG arms, respectively. At week 96, in the BIC arm, small but significantly larger increases were found in TC (*p* = 0.002), LDL-c (*p* < 0.001), and the TC/HDL-c ratio (*p* = 0.003) (Table 3) [29,30].

#### 3.2.3. Dolutegravir and Raltegravir

DTG and RAL combined with co-formulated ABC and 3TC (40–41%) or TDF and FTC (59–60%) were compared in the SPRING-2 trial. Small and nonsignificant changes over time in the fasting lipid profile were noted in the arms (Table 3) [20,31].

#### 3.2.4. Summary of Studies Comparing INSTs in Treatment-Naïve Patients

The initiation of RAL, DTG, or BIC was associated with similar and slight increases in plasma concentrations of TC, LDL-c, and HDL-c when the background of NRTI included TAF or ABC. This increase was higher than expected for the intraindividual long-term biological variation, which was established in 6% for TC, 7.8% for LDL-c, 19.9% for TG, and 7.3% for HDL-c [32]. These increases may be considered a return of a healthier state in terms of lipids, as previously described in observational studies, and they may be related to decreases in inflammation and immune activation mediated by virological suppression [5,8]. However, when RAL and DTG (no data are available for BIC) were combined with TDF, no changes or even a tendency toward reducing lipid plasma concentrations were found in the trials. These data confirm the intrinsic lipid-lowering effect of TDF shown in the TULIP trial [33]. It is noteworthy that the increases observed with RAL, DTG, and BIC were similar to those observed with DRV/r and ATZ/r combined with TDF in the ACTG 5257, reinforcing the hypolipidemic effects of TDF even when it is associated with a PI/r. However, the requirement of lipid-lowering therapy with the three INSTIs was exceedingly low, suggesting the low clinical relevance of the mentioned increases.

### 3.3. Switch Studies from a PI or NNRTI-Based Regimen to a Regimen That Includes an INSTI in Virologically Suppressed HIV-Infected Patients

Dyslipidemia may be an adverse event following some ART drugs, mainly ritonavir- or cobicistat-boosted regimens or EFV. The switch to an INSTI may be an option to improve lipid disturbances. We reviewed RCTs that included participants who were virologically suppressed for more than 3 months on a stable combined ART regimen.

#### 3.3.1. Raltegravir

In the SPIRAL study, patients treated with PI/r (mainly lopinavir/r (44%) and ATZ/r (35%) were randomised to switch to RAL or continue on a PI/r-based regimen while maintaining the same NtRTIs. At week 48, significant reductions in TC, LDL-c, HDL-c, TG, and the TC/HDL-c ratio in the RAL arm were compared with predominant increases in the PI/r arm (*p* < 0.001 for all comparisons except TC/HDL-c, where *p* < 0.05). The percentage of participants on lipid-lowering therapy at week 48 was double that of the RAL (12%) arm in the PI/r arm (24%) (Table 4) [34].

#### 3.3.2. Elvitegravir

In the STRATEGY-PI trial, patients on a PI/r-based regimen (ATZ/r (41%), DRV/r (39.9%), or lopinavir/r (16.6%) plus TDF and FTC were randomised to switch to co-formulated EVG/c, TDF, and FTC or continue on the PI/r-based regimen. At week 48, in the overall group, only TG plasma levels decreased significantly in the EVG/c arm compared with the PI/r arm (*p* = 0.001). However, lipid changes differed depending on the baseline PI/r. In patients that switched from ATZ/r, a mean (SD) change in the level of TG of −31.9 (207.1) was observed in patients who switched to EVG/c compared with those who continued on ATZ/r (5.3 (80.5); *p* = 0.014). By contrast, in patients who switched from DRV/r, there was a significant increase in HDL-c (1.9 (9.3)) and a decrease in TC/HDL-c ratio (−0.2 (0.78)) for those who changed to EVG/c compared with those who continued on DRV/r (−1.2 (8.1); *p* = 0.03 and 0 (0.62); *p* = 0.029, respectively). Finally, in participants who switched from lopinavir/r, there was a significant decrease in TC of 23.9 (27), TG of 59.3 (55.7), and HDL-c of 1.9 (8.9) in those who changed to EVG/c compared with those who continued on lopinavir/r (TC 1.2 (22.4); *p* = 0.002, TG-0.9 (78.8); *p* = 0.003 and HDL 6.2 (9.3); *p* = 0.016) (Table 4) [35].

In the STRATEGY-NNRTI trial, patients on an NNRTI-based regimen (EFV (77.9%), nevirapine (17%), or rilpivirine (4.4%)) plus TDF and FTC were randomised to switch to co-formulated EVG/c, TDF, and FTC or to continue in the NNRTI-based regimen. At week 48, in the overall group, only HDL-c plasma levels decreased significantly in the EVG/c arm compared with those who continued in the NNRTI-based regimen (*p* = 0.001). Lipid changes differed depending on the baseline NNRTI. In patients switching from EFV to the EVG/c-based regimen, there were mean decreases in TC of 6.9 (31.3), LDL-c of 3.9 (26.6), and HDL-c of 3.1 (9.3) compared with those who continued on EFV (TC 1.2 (22.4), *p* = 0.01, LDL-c 3.9 (21.2), *p* = 0.001 and HDL-c 0 (9.3), *p* = 0.008). By contrast, in participants switching from nevirapine or rilpivirine to EVG/c, there were significant increases in LDL-c of 8.1 (25.9) and the TC/HDL-c ratio of 0.4 (1.42) compared with those who continued with nevirapine or rilpivirine (LDL-c −3.9 (23.9), *p* = 0.018 and TC/HDL-c ratio −0.1 (0.62). *p* = 0.026) (Table 4) [36]. 

Participants remaining in treatment with ATZ/r plus TDF and FTC from the WAVES study [18] were randomised to change to the co-formulated EVG/c, TAF, and FTC or to continue with ATZ/r, TDF and FTC. At week 48, the median TC, LDL-c, and HDL-c values increased in EVG/c and remained stable in the ATZ/r arm (*p*-values of the comparison <0.001, 0.002, and 0.009, respectively). No differences were observed for TG or the TC/HDL ratio between the arms. Two participants started lipid-lowering therapy in the EVG/c arm, whereas none underwent lipid-lowering therapy in the ATZ/r arm (Table 4) [37].

#### 3.3.3. Dolutegravir

In the NEAT022 study, participants on a stable triple therapy regimen consisting of a PI/r (DRV/r (51.2%), ATZ/r (36.5%), lopinavir/r (8.7%)) plus two NtRTIs, aged ≥50 years or ≥18 years with a Framingham cardiovascular disease (CVD) 10-year risk score >10%, were randomised to switch to DTG (DTG-immediate) or remain on PI/r, while maintaining the same two NtRTIs. At week 48, patients remaining on the PI/r were switched to DTG (DTG-deferred) and followed up to 96 weeks. At week 48, median TC, LDL-c, TG, and TC/HDL-c ratio decreased in the DTG-immediate arm compared with the DTG-deferred arm (*p* < 0.001 for all comparisons). No changes were observed in HDL-c in any arm. At week 96, TC, LDL-c, TG, and the TC/HDL-c ratio decreased from baseline at similar levels in both arms (*p* > 0.05) (Table 4) [38].

#### 3.3.4. Bictegravir

In the present trial, participants on a stable triple therapy regimen consisting of a cobicistat or ritonavir-PI regimen (DRV (55.1%) and ATZ (44.8%)) plus either TDF and FTC (84%) or ABC and 3TC (15%) were randomised to switch to co-formulated BIC, TAF, and FTC or to remain on their baseline boosted-PI regimen. At week 48, TG and the TC/HDL-c ratio decreased in the BIC arm compared with the overall boosted-PI arm (*p* = 0.002 and *p* = 0.033, respectively). However, lipid changes differed depending on the baseline background of NtRTI. Small and nonsignificant changes were observed in participants who switched to the BIC arm from a boosted-PI regimen containing TDF and FTC. However, in participants switching from an ABC and 3TC regimen, the switch to BIC, TAF, and FTC was associated with significant decreases in TC (*p* = 0.0002), LDL-c (*p* = 0.001), TG (*p* = 0.0001), and the TC/HDL-c ratio (*p* = 0.012) than continuing with the boosted-PI (Table 4) [39]. 

#### 3.3.5. Summary of Switching Studies from a PI or NNRTI-Based Regimen to a Regimen That Includes an INSTI in Virologically Suppressed HIV-Infected Patients

The switch from a PI/r-based regimen to a RAL- or DTG-based regimen while maintaining the same background NtRTI was associated with significant decreases in TC, LDL-c, TG, and the TC/HDL-c ratio. By contrast, the switch from a PI/r-based regimen to EVG/c, both in combination with TDF and FTC, was associated with a significant decrease only in TG. The switch from an NNRTI to EVG/c, maintaining TDF plus FTC, was associated with changes in lipids that differed depending on the baseline NNRTI, with beneficial effects only when it was an EFV. Finally, the effect on lipids by switching from a PI-based regimen to the co-formulated BIC, TAF, and FTC depended on whether participants were undergoing regimens containing TDF or ABC at baseline. When the baseline regimen contained TDF, the switch to BIC resulted in no change in any fasting lipid parameter at week 48, indicating that the effect of switching away from TDF was balanced by switching from PI/r to BIC. By contrast, in participants on regimens containing ABC plus 3TC at baseline, the switch to BIC was associated with significant improvements in TC, LDL-c, TG, and the TC/HDL ratio, suggesting a neutral lipid profile for both BIC and TAF. 

### 3.4. Switch Studies on INSTIs in Virologically Suppressed HIV-Infected Patients 

The present study included women undergoing treatment with EVG/c plus TDF and FTC (42%) or TAF and FTC (53%); 5% underwent treatment with ATV/r plus TDF and FTC. Patients were randomised to switch to the co-formulated BIC plus TAF and FTC or to continue on the baseline regimen. Overall, at week 48, changes from baseline were similar and small in both arms for TC, LDL-c, HDL-c, and TC/HDL-c. Only the median TG change at week 48 differed between arms, favouring the BIC arm (*p* < 0.001). Similar percentages of participants started a lipid-lowering therapy in both arms, with 2% in BIC and 4% in those who continued with the baseline regimen (Table 5) [40]. 

Two RCTs assessed the switch of a regimen based on DTG to a regimen based on BIC [41,42]. In the first study, participants on treatment with DTG, ABC, and 3TC were randomised to switch to co-formulated BIC, TAF, and FTC or remain on the baseline regimen. At week 48, the median change from baseline in TC, LDL-c, HDL-c, and the TC/HDL-c ratio were small and similar between groups. The median TG change at week 48 favoured the BIC arm (*p* = 0.028). One per cent of patients started lipid-lowering therapy in the BIC arm, and 4% started in the DTG arm (*p* = 0.033) [41]. In the second study, participants underwent treatment with DTG plus either TDF and FTC (32%) or TAF and FTC (68%), and they could have documented/suspected NRTI resistance. Participants were randomised to switch to co-formulated BIC, TAF, and FTC or DTG plus TAF and FTC. There were no changes in lipid plasma levels at week 48 in any arm. At baseline, 21% of participants in each group underwent lipid-lowering therapy, 5% in the BIC and 3% in the DTG arm started during the study (*p* = 0.52) (Table 5) [42].

In summary these studies demonstrated similar and neutral lipid profiles for DTG and BIC, whereas BIC was superior to EVG/c only for TG.

### 3.5. Studies Comparing Triple Therapy and Dual Therapy That Include an INSTI in Naïve and in Virologically Suppressed HIV-Infected Patients 

In recent years, with the aim of reducing long-term toxicity, potential drug interactions, and the pill burden, regimens comprising two antiretroviral drugs (dual therapy) have been evaluated in naïve and virologically suppressed scenarios.

In the GEMINI 1 and 2 trials, naïve participants were randomised to receive a dual therapy with DTG plus 3TC or a triple therapy with DTG, TDF, and FTC. At week 48, TC, LDL-c, and TG increased in the two-drug regimen arm (adjusted mean changes: 12.4, 6.6, and 2.6, respectively) and decreased in the three-drug regimen arm (−5.8, −5.4, and −7.1, respectively), with the differences between groups being significant for each variable (*p* < 0.0001 for TC and LDL-c, *p* = 0.0457 for TG). A significantly greater increase was observed in HDL-c in the dual regimen than in the triple regimen group (5.8 vs. 0.8; *p* < 0.0001). A greater decrease in the TC/HDL-c ratio was observed in the triple regimen group (−0.24 vs. −0.12; *p* = 0.0182) [43]. Mean changes at week 96 were similar to those reported at week 48 (*p* < 0.05 for all comparisons) [44].

In two RCTs that included virologically suppressed patients undergoing treatment with 3- or 4-ART drugs, participants were randomised to continue the same regimen or switch to a co-formulated, dual therapy DTG and 3TC [45,46]. In the TANGO trial, participants were administered 3- or 4-ART drugs that included TAF and FTC (INSTI 78% (EVG/c 65%), NNRTI 13%, boosted-PI 8%). At week 48, TC, LDL-c TC/HDL-c, and TG decreased in the dual therapy arm compared with patients that continued on the 3- or 4-drug regimen (all *p* < 0.001, except the TC/HDL-c ratio at *p* = 0.017) [45]. In the SIMPL’HIV trial, the baseline regimen included an INSTI (64%), an NNRTI (27%), or a boosted-PI (6%) plus 2 NtRTI. At week 48, in the dual therapy arm, there was a slight decrease in TC of 9.3 (24), LDL-c of 6.2 (22), and TG of 9.7 (81.4), but this decrease was also observed in the 3- or 4-drug-based regimen (−5 (27) for TC, 0.4 (23), LDL-c, and −12.4 (69) for TG) without significant differences between arms (*p* > 0.05) (Table 6) [46]. 

In the SWORD trial, participants on a stable regimen consisting of two NtRTIs (TDF 70%) plus a third drug (NNRTI 54% (36% efavirenz)), INSTI (20%), or boosted PI (26%) were randomised to continue the same regimen or switch to DTG plus rilpivirine. At week 48, the switch to DTG and rilpivirine had no effect on plasma concentrations of TC, LDL-c, HDL-c, TG, or the TC/HDL-c ratio (Table 6) [47].

In summary, a small increase in lipids was observed with DTG and 3TC in contrast to the decrease observed with triple therapy due to the intrinsic lipid-lowering effect of TDF [33]. In simplification strategies, including INSTI, a slight but favourable lipid change in participants switching to DTG plus 3TC was observed. However, no change in lipids was observed in the SWORD trial, despite the withdrawal of TDF in a high percentage of participants that reinforced the neutral effect on serum lipids of the DTG plus rilpivirine combination. 

### 3.6. Assessment of Other Lipoprotein-Related Atherogenic Biomarkers 

LDL-c is the main goal of lipid-lowering therapy for CV risk prevention. However, the qualitative properties of LDL-c are known to add information in the assessment of CV risk. LDL consists of multiple subclasses that differ in size, density, lipid, and apolipoprotein composition, metabolic behaviour, and correlation with CV risk. The predominance of small and dense LDL particles, the so-called LDL phenotype B, is reported in lipid disorders, obesity, and type 2 diabetes, and is considered a relevant risk factor for atherogenesis and coronary heart disease [48,49,50]. Oxidized lipoproteins, both LDLox and HDLox, have atherogenic properties [51]. Three of the RCTs described previously reviewed atherogenic biomarkers of lipoproteins. In the ACTG 5260s, a CV sub-study of ACTG A5257 [14], at week 96, an increase in LDLox was observed in RAL and both PI/r arms, whereas LDL particle numbers declined only in the RAL arm, and no changes were observed in LDL particle size in any arm. HDLox decreased, and the HDL particle number increased in both PI/r arms [52]. In the SPIRAL [36] and NEAT [40] studies, the LDL particle phenotype improved (increase in LDL size and decrease in the percentage of subjects with LDL phenotype B) at week 48 with the switch of PI/r to RAL and DTG, respectively [53,54]. 

## 4. Conclusions

In conclusion, RAL, DTG, and BIC show a superior lipid profile compared with PI/r, EFV, and EVG/c, in studies conducted in naïve participants, and the three INSTIs are associated with a clinically significant benefit in lipoproteins in switching strategies. Although the decrease in switching studies is shown to be lower than that observed with lipid-lowering therapy [55], it may be enough to preserve a healthy lipid profile in a high proportion of patients and contribute to preventing and reducing the increased CV risk observed in PLWH. 

## Figures and Tables

**Table 1:** Antiretroviral drugs approved in Spain up to May 2021.

Nucleoside/Nucleotide Reverse Transcriptase Inhibitors (NtRTI)	Protease Inhibitors (PI)
Zidovudine Lamivudine (3TC) Emtricitabine (FTC) Abacavir (ABC) Tenofovir disoproxil fumarate (TDF) Tenofovir alafenamide (TAF) Didanosine * Stavudine *	Lopinavir/r Atazanavir/r (ATZ/r) Fosamprenavir/r Darunavir/r (DRV/r) Darunavir/c (DRV/c) Saquinavir/r * Indinavir * Nelfinavir * Tipranavir/r *
**Non-Nucleoside Reverse** **Transcriptase Inhibitors (NNRTI)**	**Entry Inhibitors**
Nevirapine Efavirenz (EFV) Etravirine Rilpivirine Doravirine	Enfuvirtide Maraviroc
**Integrase Strand Transfer Inhibitor (INSTI)**	
Raltegravir (RAL) Elvitegravir/c (EVG/c) Dolutegravir (DTG) Bictegravir (BIC)	

* Drugs not further used. r: ritonavir; c cobicistat.

**Table 3:** Comparison of different INSTIs in treatment-naïve patients.

Trial, Year [Ref] Design of the Study	Treatment *n* (Participants); Age; % Men	Study Period	Total Cholesterol	LDL-c	HDL-c	Triglycerides	TC/HDL-c	*N* (%) on Lipid-Lowering Therapy
2015 [26] Double-blind	**EVG/c, TDF, FTC** *n* = 867; 35; 85%	Baseline ^1^ Week 48 ^2^	163 14	104 5	44 4	100 8	3.6 0.1	25 (2.9%)
	**EVG/c, TAF, FTC** *n* = 866; 33; 85%	Baseline ^1^ Week 48 ^2^	160 29 *	101 14 *	44 7 *	95 19 *	3.6 0.1	31 (3.6%)
**GS-US-380-1490** 2017 [27] 2019 [28] Double-blind	**DTG, TAF, FTC** *n* = 325; 34; 89%	Baseline ^3^	161 (138,186)	99 (82,124)	43 (35,52)	95 (70,131)	3.7 (3.1,4.5)	
		Week 48 ^4^	15 (1,31)	12 (−3,25)	5 (−1,12)	7 (−14,35)	−0.1 (−0.6,0.4)	6 (2%)
		Week 96 ^4^	16 (−2,34)	16 (0,32)	5 (−1,12)	6 (−17,32)	−0.1 (−0.6,0.5)	12 (4%)
	**BIC, TAF, FTC** *n* = 320; 33; 88%	Baseline ^3^	156 (136,182)	98 (81,120)	43 (35,52)	97 (72,134)	3.7 (3,4.5)	
		Week 48 ^4^	12 (−3,30)	9 (−6,25)	5 (0,11)	3 (−21,31)	−0.1 (−0.5,0.3)	5 (2%)
		Week 96 ^4^	17 (−1,35)	19 (4,36)	4 (−1,9)	6 (−17,39)	0 (−0.5,0.5)	11 (3%)
**GS-US-380-1489** 2017 [29] 2019 [30] Double-blind	**DTG, ABC, 3TC** *n* = 315; 32; 90%	Baseline ^3^	162 (138,186)	101 (84,126)	42 (35,51)	96 (66,138)	3.7 (3,4.6)	
		Week 48 ^4^	11 (−6,28)	4 (−9,18)	5 (0,11)	3 (−25,27)	−0.2 (−0.7,0.2)	9 (2.9%)
		Week 96 ^4^	8 (−7,36)	5 (−5,24)	5 (−1,12)	6 (−21,30)	−0.2 (−0.7,0.3)	
	**BIC, TAF, FTC** *n* = 314; 31; 91%	Baseline ^3^	159 (133,181)	101 (83,123)	42 (34,51)	93 (67,132)	3.7 (3,4.7)	
		Week 48 ^4^	13 (−3,31)	7 (−5,21)	5 (−2,11)	5 (−20,37)	−0.1 (−0.5,0.4) *	8 (2.5%)
		Week 96 ^4^	15 (1,34) *	17 (2,32) *	4 (−1,11)	8 (−16,38)	−0.1 (−0.5,0.5) *	
**SPRING-2** 2013 [31] Double-blind	**DTG, TDF, FTC or ABC, 3TC**; *n* = 411; 37; 85%	Baseline ^5^ Week 48 ^6^	163.8 (34) 6.9 (28)	96.8 (30) 2.9 (21)	44.4 (12) 2.7 (11)	113.8 (64) 8.6 (91)	3.9 (1) −0.04 (1)	
	**RAL, TDF, FTC or ABC**, 3TC; *n* = 411; 35; 86%	Baseline ^5^ Week 48 ^6^	160.3 (38) 9 (29)	93.4 (32) 3.3 (23)	93.4 (32) 3.3 (23)	115.9 (82) 10.1 (93)	3.8 (1) −0.1 (2)	

Only studies with baseline data are shown. * *p*-value < 0.05 for comparisons between treatment arms. Superindex numbers indicate the statistical parameters to express data: ^1^ Median; ^2^ Median change; ^3^ Median (interquartile range); ^4^ Median change (interquartile range) ^5^ Mean (standard deviation); ^6^ Mean change (standard deviation). Abbreviations: ABC, abacavir; BIC, bictegravir; DTG, dolutegravir; EVG/c, cobicistat-booster elvitegravir; FTC, emtricitabine; RAL, raltegravir; TAF, tenofovir alafenamide; TDF, tenofovir disoproxil fumarate; 3TC lamivudine.

**Table 4:** Switch studies from a PI or NNRTI-based regimen to a regimen including an INSTI in virologically suppressed HIV-infected patients.

Trial, Year [Ref] Design of the Study	Treatment *n* (Participants); Age; % Men	Study Period	Total Cholesterol	LDL-c	HDL-c	Triglycerides	TC/HDL-c	*N* (%) on Lipid Lowering Therapy
**SPIRAL** 2010 [34] Open-label	**RAL**; *n* = 139; 44; 81%	Baseline ^1^	198 (171,226)	121(97,141)	44 (35,54)	168 (117,270)		27 (19%)
		Week 48 ^2^	−22.2 (−11.2%)	−7.9 (−6.5%)	−1.4 (−3.2%)	−37.1 (−22.1%)	−4.85%	16 (12%)
	**PI/r**; *n* = 134; 45; 72%	Baseline ^1^	198(171,223)	122 (97,147)	43 (37,51)	174 (114,236)		28 (21%)
		Week 48 ^2^	+3.6 (1.8%) *	−3.5 (−2.9%) *	+2.5 (5.8%) *	+8.2 (4.7%) *	−1.28% *	32 (24%)
**STRATEGY-PI** 2014 [35] Open-label	**EVG/c, TDF, FTC** *n* = 293; 41; 85%	Baseline ^3^	186.9 (39)	120.8 (34.4)	50.9 (15.4)	153.1 (154.9)	3.9 (1.24)	
		Week 48 ^4^	−4.4 (62.8)	−1.2 (22.8)	1.2 (8.5)	−29.2 (143.4)	−0.1 (0.8)	
	**PI/r, TDF, FTC** *n* = 140; 40; 86%	Baseline ^3^	189.9 (38.2)	123.9 (33.6)	50.2 (12.7)	145.1 (79.6)	4 (1.12)	
		Week 48 ^4^	−8.8 (68.1)	1.2 (27)	1.2 (10.8)	8.8 (74.3) *	0.2 (3.23)	
**STRATEGY-NNRTI** 2014 [36] Open-label	**EVG/c, TDF, FTC** *n* = 291; 43; 92%	Baseline ^3^	191.1 (35.5)	120.8 (30.9	54 (14.3)	140.7 (116.8)	3.7 (1.1)	
		Week 48 ^4^	−6.9 (31.3)	−3.9 (26.6)	−3.1 (9.3)	−4.4 (81.4)	0.1 (0.8)	
	**NNRTI, TDF, FTC** *n* = 143; 39; 94%	Baseline ^3^	188 (35.5)	118.1 (32)	52.9 (15.1)	141.6 (107.9)	3.8 (1.3)	
		Week 48 ^4^	1.1 (22.4)	3.9 (21.2)	0 (9.3) *	−7.9 (75.2)	0 (0.8)	
**WAVES switch** 2018 [37] Double-blind	**EVG/c, TAF, FTC** *n* = 159; 36	Baseline ^1^	171 (148,203)	105 (89,133)	50 (43,61)	105 (80,141)	3.3 (2.8,4.1)	2 (1%)
		Week 48 ^5^	27 (7,46)	16 (1,34)	5 (−1,12)	3 (−20,33)	0.1 (−0.1,0.5)	
	**ATZ/r, TDF, FTC** *n* = 53; 36	Baseline ^1^	180 (254,201)	115 (95,133)	56 (44,64)	105 (80,136)	3.2 (2.7,4.1)	0
		Week 48 ^5^	5 (−7,24) *	8 (−10,18) *	0 (−4,7) *	11 (−9,41)	0 (−0.3,0.4)	
**NEAT022** 2018 [38] Open-label	**DTG-immediate** *n* = 205; 54; 88.3%	Baseline ^1^	201 (174,223)	120 (97,143)	46 (39,58)	142 (106,204)	4.2 (3.4,5.4)	
		Week 48 ^2^	−17.5 (8.7%)	−9.2 (7.7%)	0.5 (1.1%)	−26.1 (18.4%)	−0.3 (7%)	
		Week 96 ^2^	−15.7 (7.8%)	−8.3 (6.9%)	1.3 (2.9%)	−22.2 (15.6%)	−0.3 (6.4%)	
	**DTG-deferred** *n* = 210; 53; 90%	Baseline ^1^	197 (174,216)	120 (97,139)	46 (39,58)	142 (106,195)	4.1 (3.4,5.2)	
		Week 48 ^2^	1.4 (0.7%) *	2.4 (2%) *	1.1 (2.5%)	5.9 (4.2) *	0.02 (0.4%)	
		Week 96 ^2^	−11.4 (5.8%)	−5.4 (4.5%)	1.8 (3.9%)	−17 (12.1%)	−0.3 (7%)	
2018 [39] Open-label	**BIC, TAF, FTC** **(overall group)**; *n* = 285; 48; 84%	Baseline ^1^	188 (163,215)	121 (101,148)	47 (39,55)	122 (83,176)	4 (3.3,4.9)	8 (3%)
		Week 48 ^5^	1 (−17,20)	0 (−16,15)	3 (−3,7)	−6 (−42,22)	−0.2 (−0.6,0.3)	
	**BIC, TAF, FTC** **(from ABC/3TC)**; *n* = 47; no data	Baseline ^1^	199 (178,223)	130 (113,157)	50 (40,56)	128 (87,170)	4.1 (3.4,4.9)	
		Week 48 ^5^	−11 (−31,2) *	−7 (−31,0) *	1 (−4,5)	−31 (−51,−1) *	−0.4 (−0.7,0) *	
	**PI/r or PI/c and TDF, FTC or ABC, 3TC**; *n* = 287; 47; 82%	Baseline ^1^	183 (160,214)	118 (98,143)	46 (39,57)	121 (87,163)	3.8 (3.1,4.9)	10 (3%)
		Week 48 ^5^	5 (−12,18)	3 (−14,18)	1 (−4,7)	4 (−29,38) *	0 (−0.5,0.4) *	

Only studies with baseline data are shown. * *p*-value < 0.05 for comparisons between treatment arms. Superindex numbers indicate the statistical parameters to express data: ^1^ Median (interquartile range); ^2^ Median change [percentage of change]; ^3^ Mean (standard deviation); ^4^ Mean change (standard deviation); ^5^ Median change (interquartile range). Abbreviations: ABC, abacavir; ATZ/r, ritonavir-boosted atazanavir; BIC, bictegravir; DTG, dolutegravir; DRV/r, ritonavir-boosted darunavir; EVG/c, cobicistat-booster elvitegravir; FTC, emtricitabine; PI/c, cobicistat-boosted protease inhibitor; PI/r, ritonavir-boosted protease inhibitor; RAL, raltegravir; TAF, tenofovir alafenamide; TDF, tenofovir disoproxil fumarate; 3TC lamivudine.

**Table 5:** Switch studies between INSTIs in virologically suppressed HIV-infected patients.

Trial, Year [Ref] Design of the Study	Treatment *n* (Participants); Age; % Men	Study Period	Total Cholesterol	LDL-c	HDL-c	Triglycerides	TC/HDL-c	% on Lipid Lowering Therapy
2019 [40] Open-label	**EVG/c plus TDF, FTC or TAF, FTC**; *n* = 236; 40; no data	Baseline ^1^	193 (167,225)	122 (100,149)	56 (46,68)	99 (75,137)	3.4 (2.8,4.1)	
		Week 48 ^2^	−1 (−17,16)	−1 (−14,13)	−1 (−6,5)	4 (−15,28)	0 (−0.3,0.3)	4%
	**BIC, TAF, FTC** *n* = 234; 39; no data	Baseline ^1^	196 (171,224)	120 (101,151)	56 (47,69)	105 (78,151)	3.4 (2.9,4.1)	
		Week 48 ^2^	−4 (−22,15)	−3 (−15,14)	−1 (−7,5)	−10 (−28,12) *	0 (−0.3,0.3)	2%
2019 [41] Double-blind	**DTG, ABC, 3TC** *n* = 281; 45; 90%	Baseline ^1^	186 (162,213)	118 (99,141)	48 (41,59)	111 (78,156)	3.8 (3,4.7)	1%
		Week 48 ^2^	2 (−17,18)	2 (−14,14)	0 (−4,6)	3 (−23,30)	0 (−0.5,0.4)	
	**BIC, TAF, FTC** *n* = 282; 47; 88%	Baseline ^1^	182 (162,203)	113 (95,133)	49 (40,59)	111 (76,161)	3.7 (3,4.5)	4%
		Week 48 ^2^	0 (−17,18)	1 (−13,18)	−1 (−6,4)	−5 (−34,23) *	0 (−0.4,0.4)	
2018 [42] Double-blind	**DTG, TAF, FTC** *n* = 281; 50; 85%	Baseline ^1^	179 (156,209)	107 (91,137)	44 (38,55)	130 (83,179)	3.9 (3.3,4.8)	3%
		Week 48 ^2^	−1 (−18,17)	4 (−11,17)	1 (−4,5)	0 (−26,30)	0 (−0.4,0.4)	
	**BIC, TAF, FTC** *n* = 284; 51; 86%	Baseline ^1^	179 (150,208)	107 (82,133)	46 (39,58)	117 (83,159)	3.7 (3.1,4.6)	5%
		Week 48 ^2^	−1 (−20,15)	3 (−14,19)	0 (−4,4)	1 (−30,30)	−0.1 (−0.4,0.4)	

Only studies with baseline data are shown. * *p*-value < 0.05 for comparisons between treatment arms. Superindex numbers indicate the statistical parameters to express data:^1^ Median (interquartile range); ^2^ Median change (interquartile range). Abbreviations: ABC, abacavir; BIC, bictegravir; DTG, dolutegravir; EVG/c, cobicistat-booster elvitegravir; FTC, emtricitabine; TAF, tenofovir alafenamide; TDF, tenofovir disoproxil fumarate; 3TC lamivudine.

**Table 6:** Switch studies from a triple therapy to a dual therapy that include an INSTI in virologically suppressed HIV-infected patients.

Trial, Year [Ref] Design of the Study	Treatment *n* (Participants); Age; % Men	Study Period	Total Cholesterol	LDL-c	HDL-c	Triglycerides	TC/HDL-c
**TANGO** 2020 [45] Open-label	**3–4 drug ART**, *n* = 372; 39; 91.1%	Baseline ^1^	189.2	111.9	54	132.7	3.9
		Week 48 ^2^	2.3%	6%	1.7%	6%	0.5%
	**DTG, 3TC**, *n* = 369; 40; 93.2%	Baseline ^1^	193	111.9	54	141.6	3.9
		Week 48 ^2^	−4.5% *	−5.5% *	−1.2%	−11.2% *	−3.3% *
**SWORD** 2018 [47] Open-label	**3–4 drugs ART**, *n* = 511; 43; 79%	Baseline ^1^	186.7	108.8	53.3	126.3	3.73
		Week 48 ^1^	187	107.5	54.7	125.8	3.65
	**DTG, rilpivirine**, *n* = 513; 43; 77%	Baseline ^1^	184.3	107.2	52.3	126.4	3.78
		Week 48 ^1^	186.1	109	54.1	118	3.67

In the tables, only studies with baseline data are presented. * *p*-value < 0.05 for comparisons between treatment arms. Superindex numbers indicate the statistical parameters to express data: ^1^ Mean; ^2^ Percentage change from baseline on adjusted ratio (week 48 to baseline).

## Data Availability

Not applicable.
